# A systematic review and meta-analysis of blood interleukin-4 levels concerning malaria infection and severity

**DOI:** 10.1186/s12936-022-04237-z

**Published:** 2022-07-12

**Authors:** Kwuntida Uthaisar Kotepui, Phiman Thirarattanasunthon, Pongruj Rattaprasert, Manas Kotepui

**Affiliations:** 1 Medical Technology Program, School of Allied Health Sciences, Walailak University, Tha Sala, Nakhon Si Thammarat, Thailand; 2 Public Health Program, School of Public Health, Walailak University, Tha Sala, Nakhon Si Thammarat, Thailand; 3 Department of Protozoology, Faculty of Tropical Medicine, Mahidol University, Bangkok, Thailand

**Keywords:** Severe malaria, Interleukin-4, IL-4, Cerebral malaria, Immune response

## Abstract

**Background:**

Interleukin (IL)-4 had been linked to malaria severity, but the findings are controversial, and the evidence is inconsistent and imprecise. In the current investigation, data on IL-4 levels in patients with severe and uncomplicated malaria were compiled.

**Methods:**

The systematic review was registered at PROSPERO (CRD42022323387). Searches for relevant articles on IL-4 levels in patients with severe malaria and studies that examined IL-4 levels in both uncomplicated malaria and healthy controls were performed in PubMed, Embase, and Scopus using the search strategy without limitation to publication years or language. The quality of all included studies was evaluated using The Strengthening the Reporting of Observational Studies in Epidemiology (STROBE) Statement: standards for reporting observational studies**.** Qualitative and quantitative data syntheses were performed. The random-effects model, which weights each study according to its between- and within-study variance, was used to pool the mean difference (MD) of individual studies. The degree of heterogeneity was determined using Cochran's Q and I^2^ statistics. Additionally, meta-regression and subgroup analyses were perfomed to investigate possible sources of heterogeneity. The outliers were identified using the leave-one-out method and assessed publication bias using funnel plots, Egger’s test, and a contour-enhanced funnel plot**.**

**Results:**

A total of 2300 studies were identified through database searches, and 36 were included for analyses. The meta-analysis results showed lower mean IL-4 levels in severe malaria (434 cases) than in uncomplicated malaria (611 cases) (*P* = 0.01, pooled MD: −3.36 pg/mL, 95% confidence intervals CI −5.55 to −1.16 pg/mL, *I*^*2*^: 98.15%, 11 studies). The meta-analysis results showed no difference in mean IL-4 levels between cerebral malaria (96 cases) and noncerebral severe malaria (108 cases) (*P* = 0.71, pooled MD: 0.86 pg/mL, 95% CI −3.60 to 5.32 pg/mL, *I*^*2*^ 92.13%, four studies). Finally, no difference was found in mean IL-4 levels between uncomplicated malaria (635 cases) and healthy controls (674 cases) (*P* = 0.57, pooled MD: 0.79 pg/mL, 95% CI −1.92 to 3.50 pg/mL, *I*^*2*^: 99.89%, 11 studies).

**Conclusion:**

The meta-analysis revealed lower IL-4 levels in patients with severe malaria than in those with uncomplicated malaria, though a trend toward comparable IL-4 levels between both groups was more likely because several sources of heterogeneities were observed. Based on the limited number of studies included in the meta-analysis, until additional investigations have been conducted, IL-4 consideration as an alternative prognostic factor for malaria severity is not warranted.

**Supplementary Information:**

The online version contains supplementary material available at 10.1186/s12936-022-04237-z.

## Background

An estimated 1.7 billion malaria cases and 10.6 million malaria deaths were averted globally between 2000 and 2020 [1]. Severe malaria exhibits various manifestations and frequently manifests as cerebral malaria, severe anaemia, or respiratory distress. In Africa, severe malaria and deaths were concentrated among children under age five, especially those under age three [1]. Cytokines and chemokines are elevated in the peripheral blood during acute malaria infection and contribute to parasite clearance, but they are also likely responsible for several symptoms and pathological changes observed during malaria [2]. Nevertheless, the outcome of malaria is influenced by individual variances in the inflammatory and immunological responses to the parasites. As a result, little is known about their role in the pathophysiology of life-threatening clinical symptoms. Hence, investigating how the immune systems of severe malaria patients respond to inflammatory indicators should provide insight into the underlying mechanisms that lead to either positive or negative outcomes, as well as new diagnostic markers for disease severity.

Previously, researchers discovered that TNF and Th2 group cytokines, such as IL-6 and IL-10 concentrations, were elevated in individuals with severe malaria, indicating that these cytokines play a role in the etiology of malaria disease severity [3–5]. Nevertheless, what role IL-4, a member of the Th2 group of cytokines, may play in the pathophysiology of severe malaria is unclear, and further research is required. Only a fraction of activated haematopoietic cells, including T cells, Fc epsilon R1+ mast cells, and basophils, produce IL-4, which is critical for antibody formation, haematopoiesis, inflammation, and the establishment of effector T-cell responses [6]. IL-4 is best recognized for its role in defining the Th2 phenotype of lymphocytes and regulating cell proliferation, apoptosis, and expression of several genes in various cell types, including lymphocytes, macrophages, and fibroblasts, as well as epithelial and endothelial cells [7]. Several studies investigated the IL-4 levels in different clinical malaria severity [8–22]. However, the uncertainty and controversy surrounding the role of IL-4 in malaria infection and severity exist. Thus, in the current investigation, data on IL-4 levels in patients with severe and uncomplicated malaria were compiled. The findings of this study will inform future research into IL-4 as a predictive biomarker for severe malaria.

## Methods

The systematic review was registered at PROSPERO (CRD42022323387).

### Eligibility criteria

The Participant, Intervention, Control, and Outcome (PICO) paradigm was created to develop the primary study objective for the systematic literature review. All types of studies were evaluated. Next, those that examined IL-4 levels in severe malaria, as well as studies that examined IL-4 levels in both uncomplicated malaria and healthy controls were included. However, those that enrolled only patients with uncomplicated malaria, asymptomatic malaria, pregnant women, in vitro studies, animal studies, literature reviews, conference proceedings, and abstracts without complete methodology were excluded.

### Outcomes

The primary outcome measure was the difference in IL-4 levels between patients with severe and uncomplicated malaria. The secondary outcome measure was the difference in IL-4 levels between patients with severe cerebral malaria and those with noncerebral severe malaria. Finally, the difference in IL-4 levels between patients with uncomplicated malaria and healthy control individuals served as the tertiary endpoint.

### Participants

Severe malaria is defined as the presence of one or more of the following symptoms in the absence of an identifiable alternative cause and the presence of *Plasmodium falciparum* asexual parasitaemia: impaired consciousness, prostration, multiple convulsions, acidosis, hypoglycemia, severe malarial anaemia, renal impairment, jaundice, pulmonary edema, significant bleeding, shock, and hyperparasitaemia. Severe *Plasmodium vivax* and *Plasmodium knowlesi* malaria were classified similarly to falciparum malaria, but without regard for parasite density thresholds [23]. Patients with severe malaria served as “Participants” in the primary outcome. Those with cerebral malaria served as “Participants” in the secondary outcome, and patients with uncomplicated malaria served as “Participants” in the tertiary outcome.

### Intervention

None.

### Control

Uncomplicated malaria is characterized by asexual parasitaemia but lacking any severe malaria symptoms. Patients with uncomplicated malaria served as “Control” in the primary outcome. Those with noncerebral severe malaria served as “Control” in the secondary outcome. In the tertiary outcome, healthy controls served as “Control.”

### Information sources and search strategy

Scopus, PubMed, and EMBASE searches were conducted from inception to March 21, 2022, using the search strategy "("interleukin-4" OR "interleukin 4" OR IL-4 OR IL4 OR BSF-1 OR Binetrakin OR BCGF-1 OR MCGF-2) AND (malaria OR plasmodium). The Medical Subject Headings (MeSH) were used in the search from PubMed. The literature search was not restricted by publication year, language, or sociodemographic characteristics of participants (e.g., age, sex, region). Also, relevant studies were identified through Google Scholar searches and the reference lists of the included research. Additional file 13: Table S1 details the search approach.

### Study selection and data extraction

EndNote (Clarivate Analytics, London, UK) was used to import the literature search results. The process of selecting studies began with a review of titles and abstracts gathered from various electronic databases. Then, the complete text of eligible papers was examined following the eligibility criteria. Two review authors (KUK and MK) independently screened articles for inclusion and extracted data on general information, including authors, study design, publication year, study location (year), types of participants, types of severe complications, *Plasmodium* spp., age, gender, IL-4 levels (mean with standard deviation (SD) or median with range) (pg/mL), parasite density, the method for malaria detection, methods for IL-4 measurements, brand names of IL-4 assays, and types of blood samples for IL-4 measurements to a standardized sheet. Disagreements between the two review authors were handled by requesting a conclusion from the third review author.

### Critical appraisal

The quality of all included studies was evaluated using The Strengthening the Reporting of Observational Studies in Epidemiology (STROBE) Statement: standards for reporting observational studies [24]. Each study was evaluated on 22 items; a score of 1 (yes) or 0 (no/unclear) was awarded to each item, which was then added to generate an overall quality score ranging from 0 to 100%. (Additional file 14: Table S2). Next, the research was classified into three groups based on their overall score and percentile. Studies were classified as high quality (> 75% percentile), moderate quality (50–75% percentile), or low quality (< 50% percentile).

### Data syntheses

Qualitative and quantitative data syntheses were performed. The qualitative synthesis process involved narratively describing and combining the data from each study. The quantitative synthesis entailed the statistical analysis of the pooled outcome. The statistical analysis pooled the mean difference (MD) and SD of IL-4 levels between groups of participants from individual studies. When the mean and standard deviation were unavailable, the median and range (or interquartile range, IQR) were used and converted to mean and SD, as previously described [25, 26]. If a study reported a median without a range, the median IL-4 levels were used for qualitative but not quantitative analysis. The random-effects model, which weights each study according to its between- and within-study variance, was used to pool the MDs of individual studies [27, 28]. Next, the degree of heterogeneity was determined using Cochran’s Q statistic and the I^2^ statistic. The forest plot displayed the MD and confidence intervals (CIs). To investigate possible sources of heterogeneity, meta-regression and subgroup analysis were performed. The subgroup analysis determined the pooled MD for each subgroup and the within-group heterogeneity. The relationship between study-level characteristics and the pooled MD was determined using the meta-regression analysis. The outliers were identified using the leave-one-out method, which involved iteratively rerunning the meta-analysis and removing studies. The publication bias was assessed using funnel plots, Egger's test, and a contour-enhanced funnel plot [29]. Stata, v.17, was used for data analyses (Stata Corporation, College Station, TX).

## Results

### Search results

**Fig. 1:**
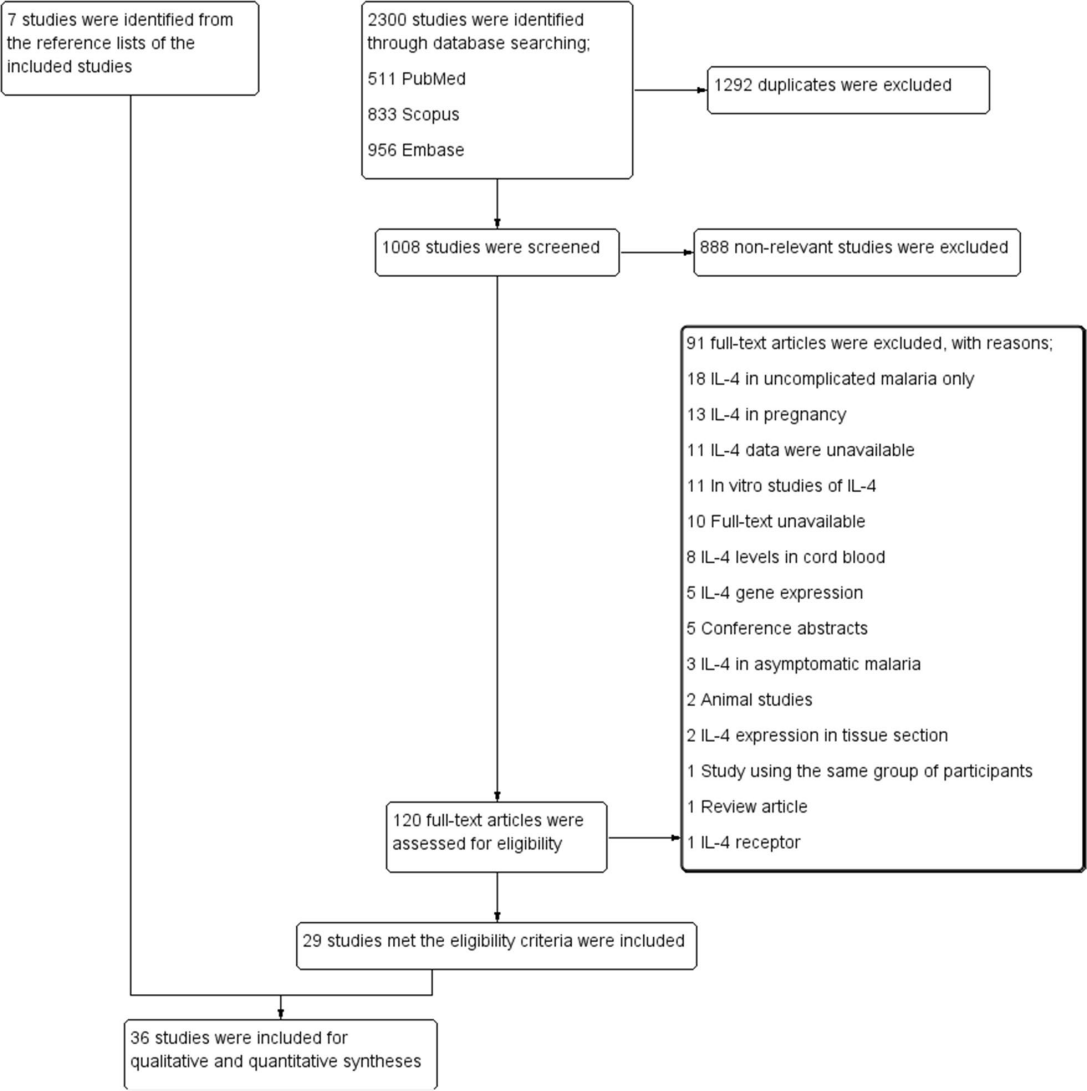
Diagram illustrating the study selection process

Through database searches, 2300 studies were identified, including 511 PubMed, 833 Scopus, and 956 Embase studies. One thousand two hundred ninety-two duplicates were eliminated, and 1008 studies were screened for titles and abstracts. After 888 studies were ruled out as irrelevant, the eligibility of 120 full-text articles was determined. Then, 92 full-text articles were excluded for the following reasons; 18 were IL-4 in uncomplicated malaria only, 13 were IL-4 in pregnancy, 12 with IL-4 data were unavailable, 11 were in vitro studies of IL-4, ten were full-text unavailable, eight reported IL-4 in cord blood, five with IL-4 gene expression, five were conference abstracts, three reported IL-4 in asymptomatic malaria, two were animal studies, two reported IL-4 in tissue section, one study using the same group of participants, one was a review, and one reported IL-4 receptor. Twenty-nine studies [8–15, 18, 20, 30–48] that met the inclusion criteria were included. From the reference lists of the included studies, seven studies [19, 21, 22, 49–52] were identified and were included in the review. Finally, 36 studies [8–15, 18–22, 30–52] were incorporated for qualitative and quantitative syntheses (Fig. 1). Among the 36 included studies, IL-4 levels in both severe and uncomplicated malaria were reported in 18 studies [19–22, 35–44, 49–52]. IL-4 levels in patients with cerebral and noncerebral severe malaria were reported in six studies [19, 21, 30, 43, 49, 52]. IL-4 levels in both uncomplicated malaria and healthy controls were reported in 24 studies [8–15, 19, 21, 22, 35–40, 43, 45–50].

### Characteristics of the included studies

Table 1 summarizes the characteristics of the studies included in the syntheses. Thirty-six studies were published between 1994 and 2021. The majority of the included studies were prospective observational studies (11, 30.6%) and cross-sectional studies (11, 30.6%). The majority of the included studies were conducted in Africa (19, 52.8%), Asia (7, 19.4%), and South America (7, 19.4%). The majority of the studies included patients infected with *P. falciparum* (26, 72.2%). The studies enrolled children (17, 47.2%), adults (10, 27.8%), and people of all ages (9, 25%). The majority of the included studies (26, 72.2%) used a single microscopic method to detect malaria parasites. Studies measured IL-4 using enzyme-linked immunosorbent assay (ELISA) (21, 58.3%) and bead-based assays (15, 41.7%). Among studies that used ELISA, most of them used Pharmingen (6, 28.6%), Genzyme (2, 9.5%), and R&D Systems (2, 9.5%). Meanwhile, among studies that used bead-based assays, most of them used Pharmingen (4, 26.7%), BioRad (3, 0.20%), Becton Dickinson Biosciences (2, 13.3%), and BioSource International (2, 13.3%). Finally, for measuring IL-4, studies used plasma (19, 52.8%) and serum (17, 47.2%). Additional file 14: Table S2 contains information about the included studies.

**Table 1:** Characteristics of 36 studies included in the study

Characteristics	N	%
Study designs		
Prospective observational studies	11	30.6
Cross-sectional studies	11	30.6
Case–control studies	7	19.4
Retrospective observational study	5	13.9
Cohort study	2	5.56
Study areas		
Africa	19	52.8
Asia	7	19.4
South America	7	19.4
Europe	3	8.33
*Plasmodium* spp.		
*P. falciparum*	26	72.2
*P. vivax*	8	22.2
*P. falciparum/P. vivax*	2	5.56
Participants		
Children	17	47.2
Adults	10	27.8
All age ranges	9	25.0
Methods for malaria detection		
Microscopy	26	72.2
Microscopy/PCR	7	19.4
Microscopy/RDT/PCR	1	2.78
PCR	1	2.78
Methods for IL-4 quantification		
ELISA	21	58.3
Pharmingen	6	28.6
Genzyme	2	9.52
R&D Systems	2	9.52
Abcam	1	4.76
Abcys	1	4.76
BioSource International	1	4.76
Diaclone	1	4.76
Euroclone	1	4.76
Medgenix	1	4.76
Ray Biotech	1	4.76
Pierce	1	4.76
Sunlong Biotech	1	4.76
T-Cell Sciences	1	4.76
Not specified brand name	1	4.76
Bead-based assays (brand names)	15	41.7
Pharmingen	4	26.7
BioRad	3	20
Becton Dickinson (BD) Biosciences	2	13.3
BioSource International	2	13.3
Millipore BV	1	6.67
LINCO Research	1	6.67
Bender MedSystems	1	6.67
Invitrogen	1	6.67
Blood sample for IL-4 quantification		
Plasma	19	52.8
Serum	17	47.2

*ELISA* Enzyme-linked immunosorbent assay, *PCR* Polymerase chain reaction, *RDT* rapid diagnostic test

### Quality of the included studies

To assess the quality of the included studies, STROBE checklists were used. The majority of the included studies were of high quality (33, 91.7%). Meanwhile, three studies [20, 32, 41] demonstrated moderate quality because of not defined study design, inadequate details of participants and statistical analysis, and lack of explanation of the limitations and implementations of the research. There was no low-quality study, and thus, all studies were included in the qualitative and quantitative syntheses (Additional file 15: Table S3).

### IL-4 levels in severe and uncomplicated malaria

The primary outcome measure was the difference in IL-4 levels between patients with severe malaria and patients with uncomplicated malaria. IL-4 levels in both severe and uncomplicated malaria were reported in 18 studies [19–22, 35–44, 49–52]. Among these 18 studies, ten studies reported higher IL-4 levels in severe malaria than in uncomplicated malaria (55.6%) [19–21, 37, 39–41, 43, 44, 50]. Meanwhile, lower IL-4 levels in severe malaria than in uncomplicated malaria were reported in five studies (27.8%) [22, 36, 38, 42, 49]. The comparable IL-4 levels between the two groups were reported in two studies (11.1%) [35, 51]. Thuma et al. [52] discovered that cerebral malaria demonstrated a higher detection rate of IL-4 (60.9%) than severe malarial anaemia (48.2%) or uncomplicated malaria (48.2%).

**Fig. 2:**
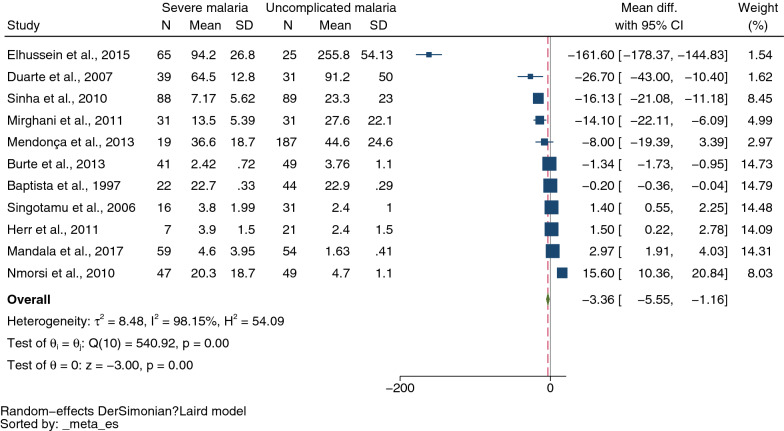
Mean difference in IL-4 levels between severe malaria and those with uncomplicated malaria patients. *CI* confidence interval; green diamond symbol, point estimate; solid line in the graph’s center at 0 effect size; The dashed red line represents the pooled mean difference between the two groups; the I^2^ value indicates the degree of heterogeneity; and p = 0.00 or less than 0.05 indicates significant heterogeneity. The weight (%) indicates the contribution of each individual result to the weighted average. IL-4 units in pg/mL

The MD of IL-4 levels between severe (434 cases) and uncomplicated malaria (611 cases) was estimated using the data from 11 studies that reported quantitative data (mean and SD, or median and range) of IL-4 levels [19–22, 35, 36, 39–41, 49, 50]. The meta-analysis results showed lower mean IL-4 levels in severe malaria than in uncomplicated malaria (*P* = 0.01, pooled MD −3.36 pg/mL, 95% CI −5.55 to −1.16) pg/mL, *I*^*2*^ 98.15%, 11 studies, Fig. 2). Due to the high degree of heterogeneity in MD across studies, meta-regression analyses were conducted using covariates study designs, continents, *Plasmodium* species, age groups, types of severe complications, malaria parasitaemia, and method and types of blood samples for IL-4 measurement. The meta-regression results showed that the regression coefficients, including study designs, types of severe complications, malaria parasitaemia, and method for IL-4 measurement, were not zero (*P* < 0.05, Additional file 16: Table S4).

The subgroup analyses of study designs, types of severe complications, and methods for IL-4 measurement were conducted. The subgroup analysis of study designs revealed no difference in IL-4 levels between severe and uncomplicated malaria between case–control studies (pooled MD −4.86 pg/mL, 95% CI −9.89 to 0.17 pg/mL, *I*^*2*^ 95.74%, four studies), prospective observational studies (pooled MD 3.25 pg/mL, 95% CI −0.18 to 6.68 pg/mL, *I*^*2*^ 95.74%, three studies), and cross-sectional studies (pooled MD −80.67 pg/mL, 95% CI −238.84 to 77.5 pg/mL, *I*^*2*^ 99.72%, two studies, Additional file 1: Fig. S1). A retrospective observational study discovered no difference in IL-4 levels between the two groups [39]; however, a cohort study revealed that patients with severe malaria demonstrated lower IL-4 levels than patients with uncomplicated malaria [21]. The subgroup analysis of types of severe complications revealed that studies that enrolled patients with cerebral and noncerebral severe malaria demonstrated lower mean IL-4 levels than patients with uncomplicated malaria (pooled MD −5.91 pg/mL, 95% CI −9.15 to −2.67) pg/mL, *I*^*2*^ 99.07%, five studies). Meanwhile, no difference was found in mean IL-4 levels between studies that enrolled patients with noncerebral severe malaria or severe complications (pooled MD −2.06 pg/mL, 95% CI −6.49 to 2.37 pg/mL, *I*^*2*^ 94.63%, four studies, Additional file 2: Fig. S2). The subgroup analysis of types of methods for IL-4 measurement revealed that studies that used ELISA demonstrated lower mean IL-4 levels in patients with severe malaria than in uncomplicated malaria patients (pooled MD −15.14 pg/mL, 95% CI −20.71 to −9.57 pg/mL, *I*^*2*^ 98.71%, seven studies). Meanwhile, no difference was found in mean IL-4 levels between the two groups of patients among studies that used bead-based assays for IL-4 measurement (pooled MD 0.53 pg/mL, 95% CI −2.37 to 3.42 pg/mL, *I*^*2*^ 95.66%, four studies, Additional file 3: Fig. S3). Next, a bubble plot between the pool MD and malaria parasitaemia was constructed, and a trend of positive correlation between a higher malaria parasitaemia and higher MD of IL-4 levels was discovered (Additional file 4: Fig. S4).

### IL-4 levels in cerebral and noncerebral severe malaria

The secondary outcome measure was the difference in IL-4 levels between patients with severe cerebral malaria and patients with noncerebral severe malaria. The difference in IL-4 levels between cerebral and noncerebral severe malaria was reported in six studies [19, 21, 30, 43, 49, 52]. Three studies reported lower IL-4 levels in cerebral malaria than in noncerebral severe malaria (50%) [21, 43, 49]. Meanwhile, in two studies, higher IL-4 levels in severe malaria than in uncomplicated malaria were reported (33.3%) [19, 52]. Armah et al. [30] reported no difference in IL-4 levels between cerebral and severe malarial anaemia. Additionally, other studies investigated IL-4 concentrations in patients with severe malaria. Biemba et al. demonstrated that cerebral malaria with severe anaemia demonstrated lower median IL-4 levels than cerebral malaria without severe anaemia (21 pg/mL vs. 64 pg/mL) in 1990–1991. Meanwhile, a study conducted in 1994 observed no difference in median IL-4 levels between the two groups (12 pg/mL) [18]. Cabantous et al. showed that IL-4 levels increased in patients with severe malaria (either CM or SA), and the highest mean IL-4 levels were observed in the T allele of IL4-33 and one copy of allele 1 of IL4 variable-number tandem repeat polymorphisms (58 pg/mL) [31]. Mohapatra et al. enrolled patients with severe malaria and showed a decrease in mean IL-4 levels compared to healthy controls (2.35 pg/mL vs. 6.06 pg/mL) [32]. Okoli et al., who enrolled patients with severe malaria, demonstrated a positive correlation between IL-4 concentrations and parasitaemia levels [33]. Thuma et al. showed that the IL-4 concentration among cerebral malaria before treatment was approximately 71–80 pg/mL [34].

**Fig. 3:**
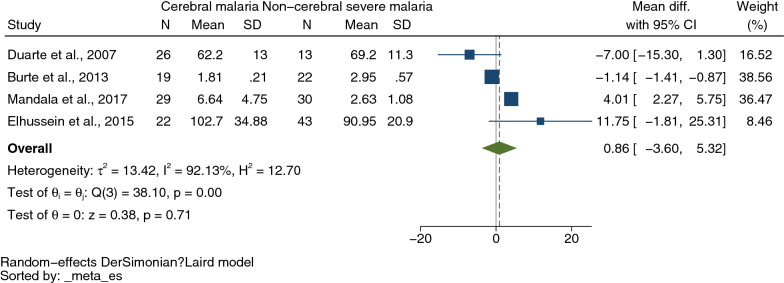
Mean difference in IL-4 levels between cerebral malaria and those with non-cerebral severe malaria patients. *CI* confidence interval; green diamond symbol, point estimate; solid line in the center of the graph at zero effect size, abbreviation; The dashed red line represents the pooled mean difference between the two groups; the I^2^ value indicates the degree of heterogeneity; and p = 0.00 or less than 0.05 indicates significant heterogeneity. The weight (%) indicates the contribution of each individual result to the weighted average. IL-4 units in pg/mL

The difference in IL-4 levels between cerebral malaria (96 cases) and noncerebral severe malaria (108 cases) was estimated using the data from four studies that reported quantitative data (mean and SD, or median and range) of IL-4 levels [19, 21, 36, 49]. The meta-analysis results showed no difference in mean IL-4 levels between cerebral malaria and noncerebral severe malaria (*P* = 0.71, pooled MD 0.86 pg/mL, 95% CI −3.60 to 5.32 pg/mL, *I*^*2*^ 92.13%, four studies, Fig. 3). The meta-regression of study designs, continents, *Plasmodium* species, age groups, malaria parasitaemia, and methods and types of blood samples for IL-4 measurement demonstrated that the regression coefficients were zero (*P* > 0.05 for all covariates, Additional file 16: Table S4). No subgroup analysis was further performed.

### IL-4 levels in uncomplicated malaria and healthy controls

The tertiary outcome was the difference in IL-4 levels between patients with uncomplicated malaria and healthy control. The difference in IL-4 levels between uncomplicated malaria and healthy controls was reported in 24 studies [8–15, 19, 21, 22, 35–40, 43, 45–50]. Higher IL-4 levels in uncomplicated malaria than in healthy controls were reported in 14 studies (58.3%) [8–10, 21, 22, 36–39, 43, 45, 47, 48, 50]. Meanwhile, lower IL-4 levels in uncomplicated malaria than in healthy controls were reported in four studies (16.7%) [11, 12, 40, 46]. The comparable IL-4 levels between the two groups were reported in six studies (25%) [13–15, 19, 35, 49].

**Fig. 4:**
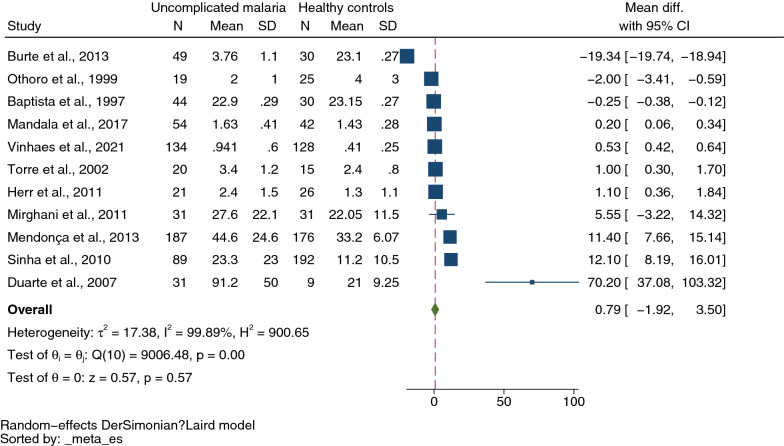
Mean difference in IL-4 levels between uncomplicated malaria patients and healthy controls. Abbreviation: CI, confidence interval; green diamond symbol, point estimate; solid line in the center of the graph at zero effect size, abbreviation; The dashed red line represents the pooled mean difference between the two groups; the I^2^ value indicates the degree of heterogeneity; and p = 0.00 or less than 0.05 indicates significant heterogeneity. The weight (%) indicates the contribution of each individual result to the weighted average. IL-4 units in pg/mL

Next, the difference in IL-4 levels between uncomplicated malaria (635 cases) and healthy controls (674 cases) was estimated from 11 studies that reported quantitative data (mean and SD, or median and range) of IL-4 levels [9, 11, 19, 21, 22, 35, 39, 40, 48–50]. The meta-analysis results showed no difference in mean IL-4 levels between uncomplicated and healthy controls (*P* = 0.57, pooled MD 0.79 pg/mL, 95% CI −1.92 to 3.50 pg/mL, *I*^*2*^ 99.89%, 11 studies, Fig. 4). Due to the high degree of heterogeneity in MD across nine studies, meta-regression analyses were conducted using the following covariates: study designs, continents, *Plasmodium* species, age groups, and methods and types of blood samples for IL-4 measurement. The meta-regression results showed that the regression coefficients, including study designs, continents, age groups, and types of blood samples for IL-4 measurement, were not zero (*P* < 0.05, Additional file 16: Table S4).

The subgroup analyses of study designs, continents, age groups, and types of blood samples for IL-4 measurement were conducted. The subgroup analysis of study designs revealed that uncomplicated malaria demonstrated higher mean IL-4 levels than healthy controls in case–control studies (pooled MD 2.79 pg/mL, 95% CI 0.70–4.87 pg/mL, *I*^*2*^ 92.88%, four studies) and a cohort study (MD 70.2 pg/mL, 95% CI 37.08–103.32 pg/mL). Meanwhile, prospective observational studies, retrospective observational studies, and cross-sectional studies showed no difference in mean IL-4 level between the two groups (MD −9.17 pg/mL, 95% CI −29.10 to 10.76 pg/mL, *I*^*2*^ 99.96%, two studies), (MD: 5.80 pg/mL, 95% CI −4.85 to 16.44 pg/mL, *I*^*2*^ 96.92%, two studies), (MD −0.98 pg/mL, 95% CI −2.67 to 0.71 pg/mL, *I*^*2*^ 83.0%, two studies, Additional file 5: Fig. S5). Subgroup analysis of age groups revealed no difference in mean IL-4 levels between the two groups among studies that enrolled only children (pooled MD −3.85 pg/mL, 95% CI −9.26 to 1.56 pg/mL, *I*^*2*^ 99.95%, four studies) and those studies that enrolled all age ranges (pooled MD: 38.40 pg/mL, 95% CI −19.03 to 95.83 pg/mL, *I*^*2*^ 91.64%, two studies). Meanwhile, higher mean IL-4 levels were observed in uncomplicated malaria than in healthy controls among studies that enrolled only adults (pooled MD 1.72 pg/mL, 95% CI 0.49–2.94 pg/mL, *I*^*2*^ 91.94%, four studies Additional file 6: Fig. S6). Subgroup analysis of continents revealed no difference in mean IL-4 levels between the two groups among studies conducted in Africa (pooled MD −3.85 pg/mL, 95% CI −9.26 to 1.56 pg/mL, *I*^*2*^ 99.95%, five studies), Asia (pooled MD 38.73 pg/mL, 95% CI −18.01 to 95.47 pg/mL, *I*^*2*^ 91.42%, two studies), and South America (pooled MD 5.80 pg/mL, 95% CI −4.85 to 16.44 pg/mL, *I*^*2*^ 96.92%). Meanwhile, higher mean IL-4 levels were observed in uncomplicated malaria than in healthy controls among studies that enrolled only adults (pooled MD 1.05 pg/mL, 95% CI −0.54 to 1.56 pg/mL, *I*^*2*^ 0%, two studies, Additional file 7: Fig. S7). Subgroup analysis of types of blood samples for IL-4 measurement revealed no difference in mean IL-4 levels between the two groups among studies that used serum (pooled MD: − 2.53 pg/mL, 95% CI: − 12.32–7.27 pg/mL, *I*^*2*^: 99.95%, five studies). Meanwhile, higher mean IL-4 levels were observed in uncomplicated malaria than in healthy controls among studies that used plasma for IL-4 measurement (pooled MD 0.97 pg/mL, 95% CI −0.03 to 1.91 pg/mL, *I*^*2*^ 97.15%, six studies, Additional file 8: Fig. S8).

### Sensitivity analysis

**Fig. 5:**
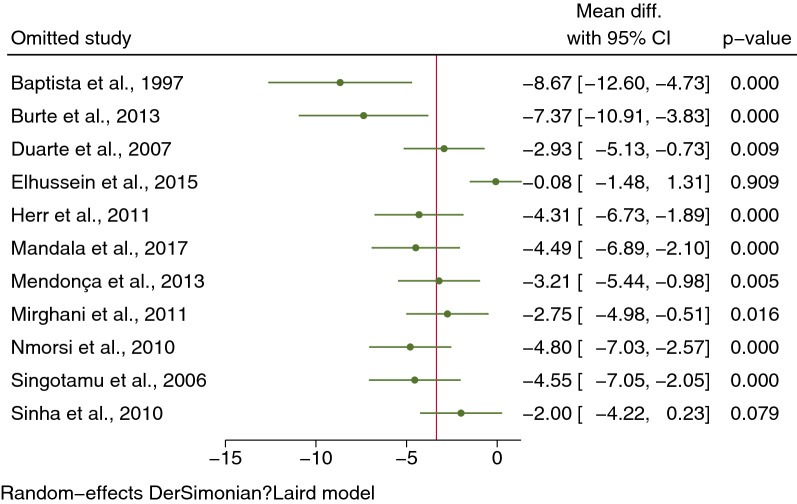
Sensitivity analysis by leave-one-out method demonstrating the differences in IL-4 levels between severe and uncomplicated malaria patients by rerunning the meta-analysis and after removing studies

The leave-one-out method was used to conduct the sensitivity analysis. When each study was excluded from the analyses, higher mean IL-4 levels were found in severe than uncomplicated malaria (*P* < 0.05), except after excluding the study by Elhussein et al. (*P* = 0.909) [36] and Singotamu et al. (*P* = 0.079) [20] from the meta-analysis (Fig. 5). However, no difference was found in IL-4 levels between cerebral malaria and noncerebral severe malaria when each study was excluded from the analyses (*P* > 0.05, Additional file 9: Fig. S9). In addition, no difference was found in IL-4 levels between uncomplicated malaria and healthy controls when each study was excluded from the analyses (*P* > 0.05), except after excluding the study by Burte et al. (*P* = 0.012) [49] from the meta-analysis (Additional file 10: Fig. S10).

### Publication bias

**Fig. 6:**
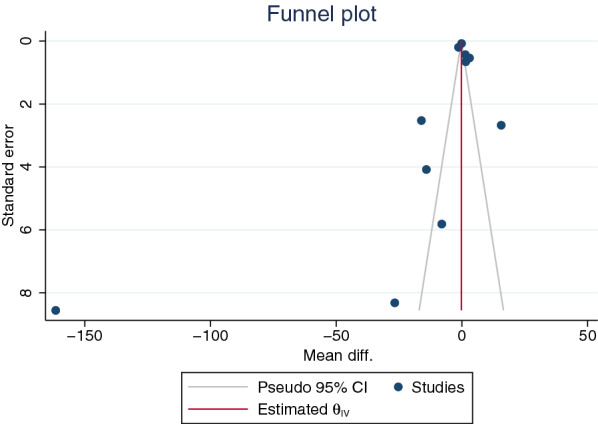
Funnel plot illustrating the distribution of the mean differences and standard error of the effect size for studies on severe and uncomplicated malaria included in the meta-analysis

**Fig. 7:**
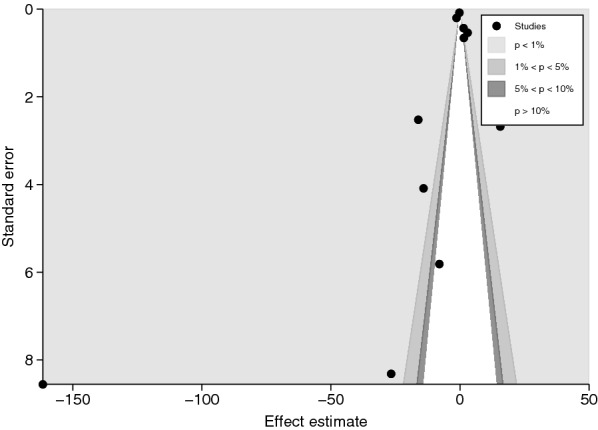
The contour-enhanced funnel plot revealed that the mean differences of IL-4 levels between severe and uncomplicated malaria patients were concentrated in a statistically significant area (*P* < 1%, 0.01), indicating that the asymmetry in the funnel plot was caused by publication bias rather than other factors

The publication bias was assessed by visualization of the funnel plot symmetry, Egger’s test, and contour-enhanced funnel plot. Results showed the funnel plot’s asymmetry that demonstrates the distribution of MDs and standard error of IL-4 among the included studies (Fig. 6). Egger’s test showed a small-study effect (*P* < 0.001). The contour-enhanced funnel plot showed that most MDs were located in significant areas (Fig. 7), indicating that the case of funnel plot asymmetry was caused by publication bias due to the small-study effect. Further, the trim and fill method was applied to determine the effect size after adjusting for the small-study effect, and the results showed that the mean IL-4 levels were lower in severe malaria than in uncomplicated malaria (pooled MD −0.246 pg/mL, 95% CI −0.0.386 to −0.106) pg/mL).

The funnel plot and the contour-enhanced funnel plot of the meta-analysis between cerebral and noncerebral severe malaria were not assessed because the number of included studies was less than 10, and the power of the tests was insufficient to distinguish chance from real asymmetry [53]. For the meta-analysis of the difference in mean IL-4 levels between uncomplicated malaria and healthy controls, results showed the funnel plot's asymmetry that demonstrates the distribution of MDs and standard error of IL-4 among the included studies (Additional file 11: Fig. S11). Egger’s test revealed a small-study effect (*P* < 0.001). Next, the contour-enhanced funnel plot showed that most MDs were located in significant areas (Additional file 12: Fig. S12), indicating that the case of funnel plot asymmetry was caused by publication bias due to the small-study effect. Further, the trim and fill method was applied to determine the effect size after adjusting for the small-study effect, and results showed that mean IL-4 levels were lower in uncomplicated malaria than in healthy controls (pooled MD −0.398 pg/mL, 95% CI −0.469 to −0.3326 pg/mL).

## Discussion

This study showed higher mean IL-4 levels in severe rather than uncomplicated malaria. Nonetheless, several sources of heterogeneity of outcomes, including study designs, types of severe complications, malaria parasitaemia, and method for IL-4 measurement, were observed. Although subgroup analyses were conducted according to study designs, types of severe complications, age groups, and heterogeneities within subgroups were still observed. These findings suggest that additional source(s) of heterogeneity may affect the difference in IL-4 levels between these two patient groups. The analysis of subgroups of study designs revealed no difference in IL-4 levels between severe and uncomplicated malaria in case–control or prospective observational studies. Nonetheless, a meta-analysis of case–control studies revealed a trend toward lower IL-4 levels in patients with severe malaria than in patients with uncomplicated malaria. On the contrary, a meta-analysis of prospective observational studies demonstrated a trend toward higher IL-4 levels in patients with severe malaria than in patients with uncomplicated malaria. Although the two study designs differ in several ways, two prospective observational studies [20, 41] were rated as demonstrating moderate quality. Thus, the trend toward lower IL-4 levels in patients with severe malaria compared to patients with uncomplicated malaria observed in case–control, cohort, and cross-sectional studies was more confident, as these studies [19, 21, 22, 35, 36, 40, 50] were of high quality.

As with the subgroup analysis of types of severe complications, the meta-analysis of studies that enrolled patients with cerebral and noncerebral severe malaria revealed a trend toward lower IL-4 levels in patients with severe malaria compared to patients with uncomplicated malaria. In contrast, the meta-analysis of noncerebral severe malaria or severe complications was not defined and found no difference in IL-4 levels between severe and uncomplicated malaria. This result indicates that lower IL-4 levels may be limited to patients with cerebral malaria, not to other severe complications. Nevertheless, a meta-analysis of cerebral and noncerebral severe malaria demonstrated no difference in IL-4 levels between the two groups, which did not support the possibility that lower IL-4 levels might be limited to patients with cerebral malaria. Furthermore, conflicting results were found about IL-4 levels between cerebral and noncerebral severe malaria. Two studies [21, 49] demonstrated a trend toward lower IL-4 levels in cerebral malaria; meanwhile, other studies [19, 36] demonstrated a trend toward higher IL-4 levels in cerebral malaria. This study showed no difference in IL-4 levels between patients with cerebral and noncerebral severe malaria, suggesting contradictory results across the included studies. As for the limited number of included studies in the meta-analysis, the meta-regression demonstrated inadequate power to identify the source of heterogeneity in each study. The result from sensitivity analysis showed no outlier, and therefore, no single study affected the direction of the meta-analysis results. In light of this study, further studies should investigate whether any difference exists in IL-4 cytokines between severe complications.

As with the subgroup analysis of the method for IL-4 measurement, the meta-analysis of studies using ELISA revealed lower IL-4 levels in patients with severe malaria compared to patients with uncomplicated malaria. On the contrary, the meta-analysis of studies using bead-based assays found no difference in IL-4 levels between patients with severe malaria and those with uncomplicated malaria. A study suggested that the two methods were comparable, but a bead-based assay could detect the bovine cytokine IL-4 and other cytokines, such as IFN-γ, IL-10, IL-12, and TNF-α, in a wider dynamic range than ELISA [54]. Addionally, ELISA is easy to use and robust for measuring single cytokines, but bead-based assays can be multiplex immunoassays to detect several cytokines in a single run [55]. Furthermore, bead-based assays exhibit 10- to 100-fold more sensitivity than ELISAs [56], suggesting that bead-based assays suit for measuring cytokines in a low concentration. Therefore, from the meta-analysis results, it might be possible that IL-4 levels between severe and uncomplicated malaria were comparable. However, no study has been conducted yet to compare the performance of the two methods for measuring IL-4, and the results of the difference remain unknown. Also, it is interesting that subgroup analysis demonstrated lower IL-4 levels in severe malaria than in uncomplicated malaria among children only, but no difference in its levels among adults. Since the acquisition of immunity to malaria is age-dependent, adults infected with malaria may develop a more robust immune response than children. Thus, a trend toward lower IL-4 levels in children with severe malaria than in those with uncomplicated malaria was clearly demonstrated. The results from the sensitivity analysis showed that Elhussein et al. [36] and Singotamu et al. [20] were outliers and further limited the robustness of the meta-analysis results. Therefore, based on the limited number of studies included in the meta-analysis, the evidence might not be strong enough to confirm the difference in IL-4 levels between the two groups. From the trim and fill analysis, it might be beneficial if further studies could investigate IL-4 as the meta-analysis filling, with missing studies showing lower IL-4 levels in patients with severe malaria than in those with uncomplicated malaria.

The current study showed no difference in IL-4 levels between patients with uncomplicated and healthy controls, indicating contradictory results across the included studies. The meta-regression results showed that study designs, age groups, continents, and types of blood samples for IL-4 measurement were sources of heterogeneities for the contradictory results. First, higher and lower IL-4 levels were observed in case–control and cohort studies. The nature of these study designs might affect the investigation of cytokine levels, particularly in case–control studies, as plasma or serum samples might be stored for a long period before measurement. For example, Thuma et al. measured IL-4 concentrations after the serum was frozen at −20 °C for 1–3 years [34]. Meanwhile, another study measured IL-4 levels in plasma after being frozen at −70 °C for several weeks or months [31]. Therefore, the difference in blood samples or storage conditions might affect the measurement of cytokines in blood samples. The subgroup analysis of blood samples showed that higher IL-4 levels in patients with uncomplicated malaria than healthy controls were limited to studies that used plasma for IL-4 measurement, but no difference was found among studies that used the serum.

Nevertheless, Duarte et al. [21], one of the included studies in the plasma subgroup, was the outlier in the sensitivity analysis and therefore affected the direction of the meta-analysis results. Second, studies conducted in Europe homogeneously showed higher IL-4 levels in uncomplicated malaria than healthy controls. This result might be because these studies enrolled only adults compared with studies conducted in other continents. This result was in accordance with results from the subgroup analysis of age groups, indicating that higher IL-4 levels in uncomplicated malaria than in healthy controls were observed only in adults. Based on the trim and fill analysis, it might be beneficial if further studies could investigate IL-4, as the meta-analysis filled with missing studies showed lower IL-4 levels in patients with uncomplicated malaria than in healthy controls.

IL-4 and IL-10 act as anti-inflammatory cytokines through a modulatory effect on IFN-γ [39]. IL-4 and IFN-γ can induce the production of antibodies in B cells, as well as attract and activate immune cells, such as macrophages and other lymphocytes, at infection sites [57, 58]. Additionally, to prevent severe malaria, IL-4 and IL-10 can directly downregulate IL-6, TNF-α, and IL-1β [59]. Basophils are the primary producers of IL-4 during parasite infection and aid in parasite clearance [60, 61]. According to a previous study, acute malaria, regardless of its severity, is characterized by elevated IL-4 levels, but these high levels significantly decrease during convalescence [19]. Furthermore, a study discovered that IL-4 was a significant predictor of hemoglobin in children with severe malarial anaemia [42]. IL-4 levels, conversely, were significantly lower in patients with severe malaria than in those with uncomplicated malaria in endemic areas compared to nonendemic areas, implying that it was a predictor of disease outcome in the endemic region [22]. In contrast, Nmorsi et al. [41] discovered that patients with severe malaria demonstrated higher IL-4 levels than patients with uncomplicated malaria. They hypothesized that elevated IL-4 levels in severe malaria could be explained by a switch from Th1 to Th2 responses during peak parasitaemia, with the latter playing a critical role in parasite clearance [41, 44, 62]. Additionally, IL-4 has been shown to induce antibody isotype switching from IgG and IgM to IgE, increasing parasite clearance [63]. Nonetheless, the *P. falciparum*-specific IgE response appears to contribute to parasite control but does not correlate with malaria protection, as functional activity was significantly greater in less severe forms than in severe or cerebral malaria [21]. Another study conducted in Thailand discovered that IL-4 levels were higher in the late stage of the disease, in contrast to the IFN-γ, which is high in the early stage of the disease [44]. Next, increased IL-4 levels were in accordance with increased IL-4 receptors in patients with severe malaria [64]. Thus, the modulation of IL-4 together with IFN-γ may indicate an alteration in the cytokine balance linked to the control of parasitaemia and the effect on clinical outcomes. A previous study supported this modulation, which showed a decreased IL4 to IFN-γ ratio upon admission and increased ratio on day 28 [44]. IL-4 levels continued to decrease on day 0 after therapy but increased on days 2, 4, 6, 8, and 10 [32].

The current study demonstrated limitations. First, significant heterogeneity occurred in the outcome among the studies included in the meta-analysis. Although the subgroup analyses of several covariates were conducted, the heterogeneity remained in an individual subgroup of all outcomes investigated. Second, publication bias occurred due to small-study effects. This bias indicated the missing results of related studies; hence, the investigation of IL-4 levels between severe and uncomplicated malaria, as well as between uncomplicated and healthy controls, is crucial for confidence in the conclusion made by the meta-analysis. Third, a limited number of studies compared the serum IL-4 levels in all outcomes, limiting the conclusion made by the current study. Alternatively, to this classic systematic review, individual patient data (IPD) meta-analysis could further provide insights for IL-4 levels in patients with malaria of varying severity.

## Conclusion

The meta-analysis demonstrated lower IL-4 levels in patients with severe malaria than in those with uncomplicated malaria, though a trend toward comparable IL-4 levels between the two groups was more likely because several sources of heterogeneity were observed. In addition, IL-4 levels between uncomplicated malaria and healthy controls were comparable, but several sources of heterogeneity, such as study designs, continents, age groups, and types of blood samples for IL-4 measurement, were needed for consideration before interpreting the results. Based on the limited number of studies included in the meta-analysis, consideration of IL-4 as an alternative prognostic factor for malaria severity is not warranted until additional investigations have been conducted.

## Supplementary Information

**Additional file 1: Figure S1. **Mean difference in IL-4 levels between severe malaria and those with uncomplicated malaria patients according to study designs. Abbreviation: CI, confidence interval; green diamond symbol, point estimate; solid line in the graph's center at 0 effect size; The dashed red line represents the pooled mean difference between the two groups; the I2 value indicates the degree of heterogeneity; and p = 0.00 or less than 0.05 indicates significant heterogeneity. The weight (%) indicates the contribution of each individual result to the weighted average. IL-4 units in pg/mL

**Additional file 2: Figure S2. **Mean difference in IL-4 levels between severe malaria and those with uncomplicated malaria patients according to types of severe complications. Abbreviation: CI, confidence interval; green diamond symbol, point estimate; solid line in the graph's center at 0 effect size; The dashed red line represents the pooled mean difference between the two groups; the I2 value indicates the degree of heterogeneity; and p = 0.00 or less than 0.05 indicates significant heterogeneity. The weight (%) indicates the contribution of each individual result to the weighted average. IL-4 units in pg/mL.

**Additional file 3: Figure S3. **Mean difference in IL-4 levels between severe malaria and those with uncomplicated malaria patients according to methods for IL-4 measurement. Abbreviation: CI, confidence interval; green diamond symbol, point estimate; solid line in the graph's center at 0 effect size; The dashed red line represents the pooled mean difference between the two groups; the I2 value indicates the degree of heterogeneity; and p = 0.00 or less than 0.05 indicates significant heterogeneity. The weight (%) indicates the contribution of each individual result to the weighted average. IL-4 units in pg/mL.

**Additional file 4: Figure S4. **Bubble plot demonstrating a trend of positive between malaria parasitemia and MD of IL-4 levels.

**Additional file 5: Figure S5. **Mean difference in IL-4 levels between uncomplicated malaria patients and healthy controls according to study designs. Abbreviation: CI, confidence interval; green diamond symbol, point estimate; solid line in the center of the graph at zero effect size, abbreviation; The dashed red line represents the pooled mean difference between the two groups; the I2 value indicates the degree of heterogeneity; and p = 0.00 or less than 0.05 indicates significant heterogeneity. The weight (%) indicates the contribution of each individual result to the weighted average. IL-4 units in pg/mL.

**Additional file 6: Figure S6. **Mean difference in IL-4 levels between uncomplicated malaria patients and healthy controls according to age groups. Abbreviation: CI, confidence interval; green diamond symbol, point estimate; solid line in the center of the graph at zero effect size, abbreviation; The dashed red line represents the pooled mean difference between the two groups; the I2 value indicates the degree of heterogeneity; and p = 0.00 or less than 0.05 indicates significant heterogeneity. The weight (%) indicates the contribution of each individual result to the weighted average. IL-4 units in pg/mL.

**Additional file 7: Figure S7. **Mean difference in IL-4 levels between uncomplicated malaria patients and healthy controls according to study sites (continents). Abbreviation: CI, confidence interval; green diamond symbol, point estimate; solid line in the center of the graph at zero effect size, abbreviation; The dashed red line represents the pooled mean difference between the two groups; the I2 value indicates the degree of heterogeneity; and p = 0.00 or less than 0.05 indicates significant heterogeneity. The weight (%) indicates the contribution of each individual result to the weighted average. IL-4 units in pg/mL.

**Additional file 8: Figure S8 **Mean difference in IL-4 levels between uncomplicated malaria patients and healthy controls according to types of blood samples for IL-4 measurement. Abbreviation: CI, confidence interval; green diamond symbol, point estimate; solid line in the center of the graph at zero effect size, abbreviation; The dashed red line represents the pooled mean difference between the two groups; the I2 value indicates the degree of heterogeneity; and p = 0.00 or less than 0.05 indicates significant heterogeneity. The weight (%) indicates the contribution of each individual result to the weighted average. IL-4 units in pg/mL.

**Additional file 9: **Figure S9. Sensitivity analysis by leave-one-out method demonstrating the differences in IL-4 levels between cerebral and non-cerebral severe malaria patients by rerunning the meta-analysis and after removing studies.

**Additional file 10: **Figure S10. Sensitivity analysis by leave-one-out method demonstrating the differences in IL-4 levels between uncomplicated malaria patients and healthy controls by rerunning the meta-analysis and after removing studies.

**Additional file 11:** Figure S11. Funnel plot illustrating the distribution of the mean differences and standard error of the effect size for studies on uncomplicated malaria patients and healthy controls included in the meta-analysis.

**Additional file 12: **Figure S12. The contour-enhanced funnel plot revealed that the mean differences of IL-4 levels between uncomplicated malaria patients and healthy controls were located in a statistically significant area (*P *< 1%, 0.01), indicating that the asymmetry in the funnel plot was caused by publication bias rather than other factors.

‹

**Additional file 13: Table S1. **Search terms.

**Additional file 14: Table S2. **Details of the included studies

**Additional file 15: Table S3. **Quality of the included studies

**Additional file 16: Table S4. **Meta-regression results.

## Abbreviations

CI: Confidence intervals
ELISA: Enzyme-linked immunosorbent assay
IPD: Individual patient data
MD: Mean difference
MeSH: Medical Subject Headings
PICO: Participant, Intervention, Control, and Outcome
SD: Standard deviation

## Declarations

### Availability of data and materials

All data and related materials are presented in this manuscript.
