# Supplementary figures and images for: A systematic review and meta-analysis of blood interleukin-4 levels concerning malaria infection and severity
Source: Malar J. 2022 Jul 12;21:217. doi: 10.1186/s12936-022-04237-z (PMC9277793; doi:10.1186/s12936-022-04237-z)

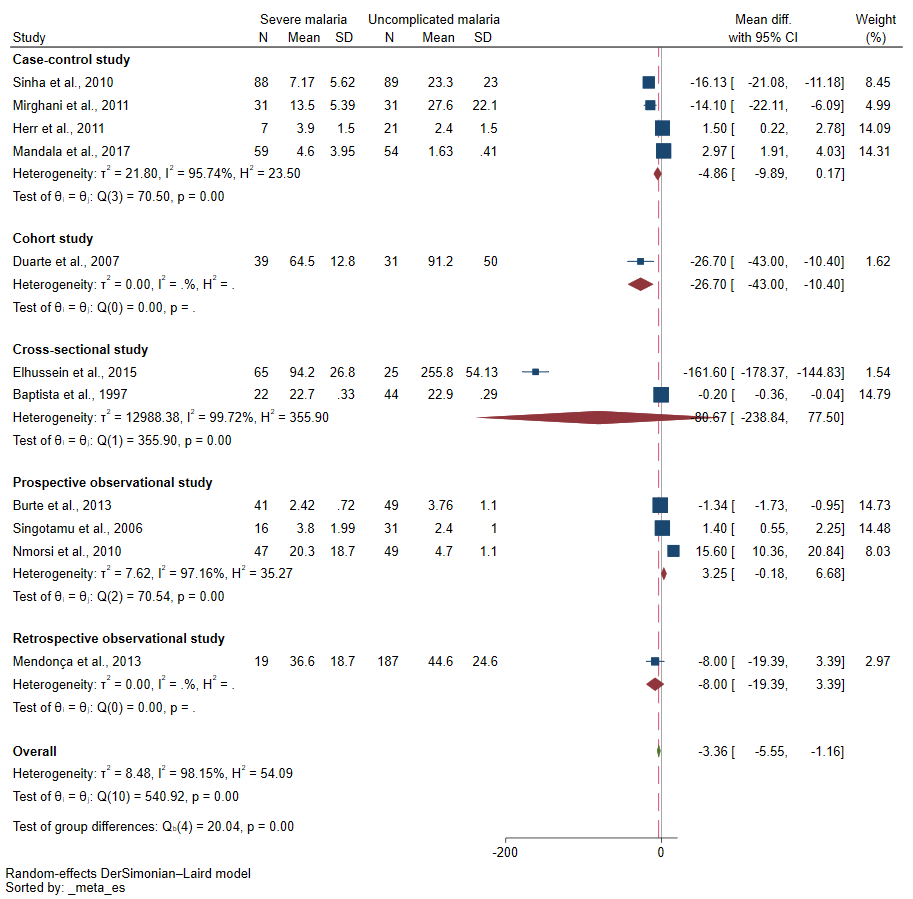

Supplement: Supplementary file 1 — Additional file 1: Figure S1. Mean difference in IL-4 levels between severe malaria and those with uncomplicated malaria patients according to study designs. Abbreviation: CI, confidence interval; green diamond symbol, point estimate; solid line in the graph's center at 0 effect size; The dashed red line represents the pooled mean difference between the two groups; the I2 value indicates the degree of heterogeneity; and p = 0.00 or less than 0.05 indicates significant heterogeneity. The weight (%) indicates the contribution of each individual result to the weighted average. IL-4 units in pg/mL [file 12936_2022_4237_MOESM1_ESM.tif]

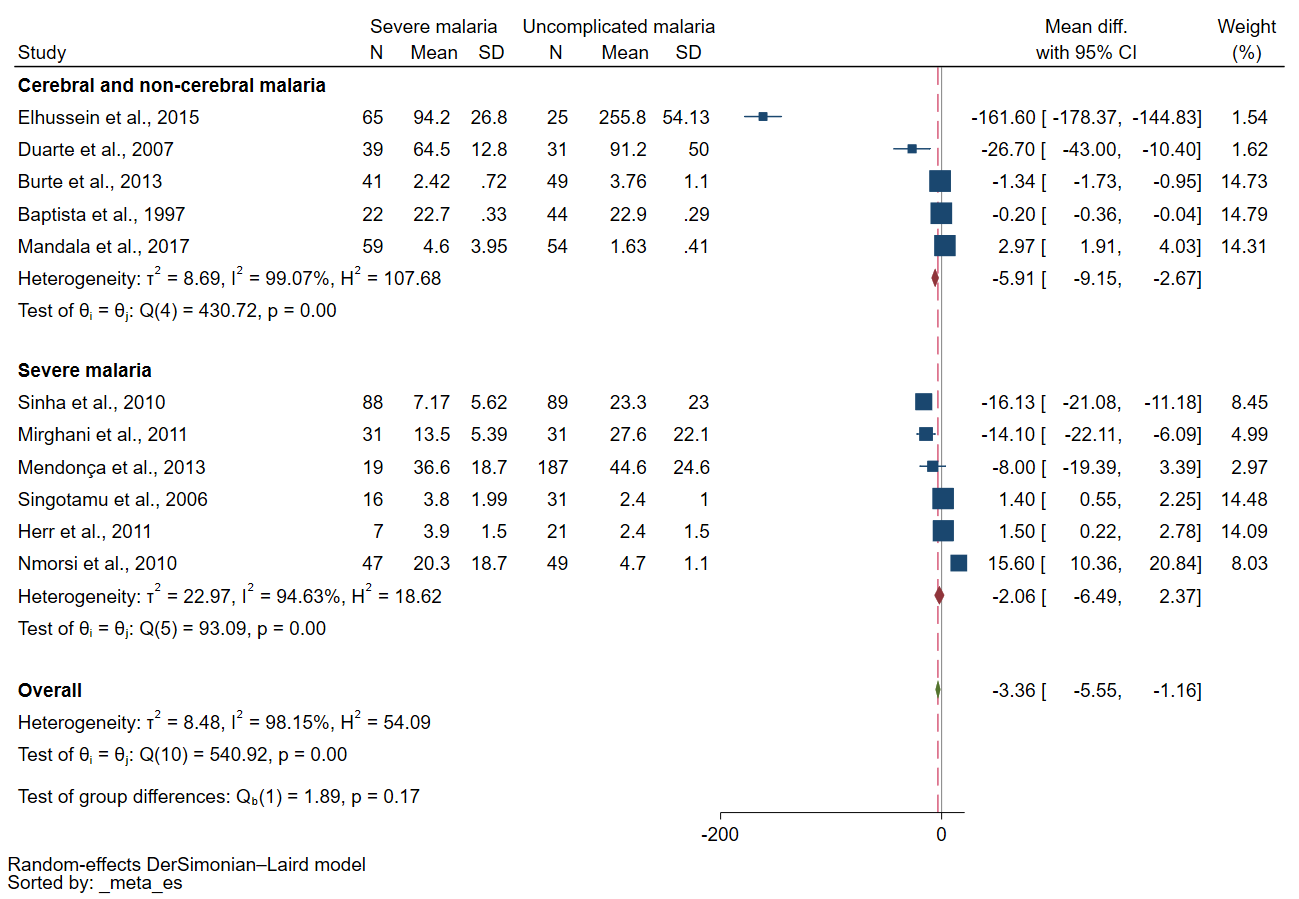

Supplement: Supplementary file 2 — Additional file 2: Figure S2. Mean difference in IL-4 levels between severe malaria and those with uncomplicated malaria patients according to types of severe complications. Abbreviation: CI, confidence interval; green diamond symbol, point estimate; solid line in the graph's center at 0 effect size; The dashed red line represents the pooled mean difference between the two groups; the I2 value indicates the degree of heterogeneity; and p = 0.00 or less than 0.05 indicates significant heterogeneity. The weight (%) indicates the contribution of each individual result to the weighted average. IL-4 units in pg/mL. [file 12936_2022_4237_MOESM2_ESM.tif]

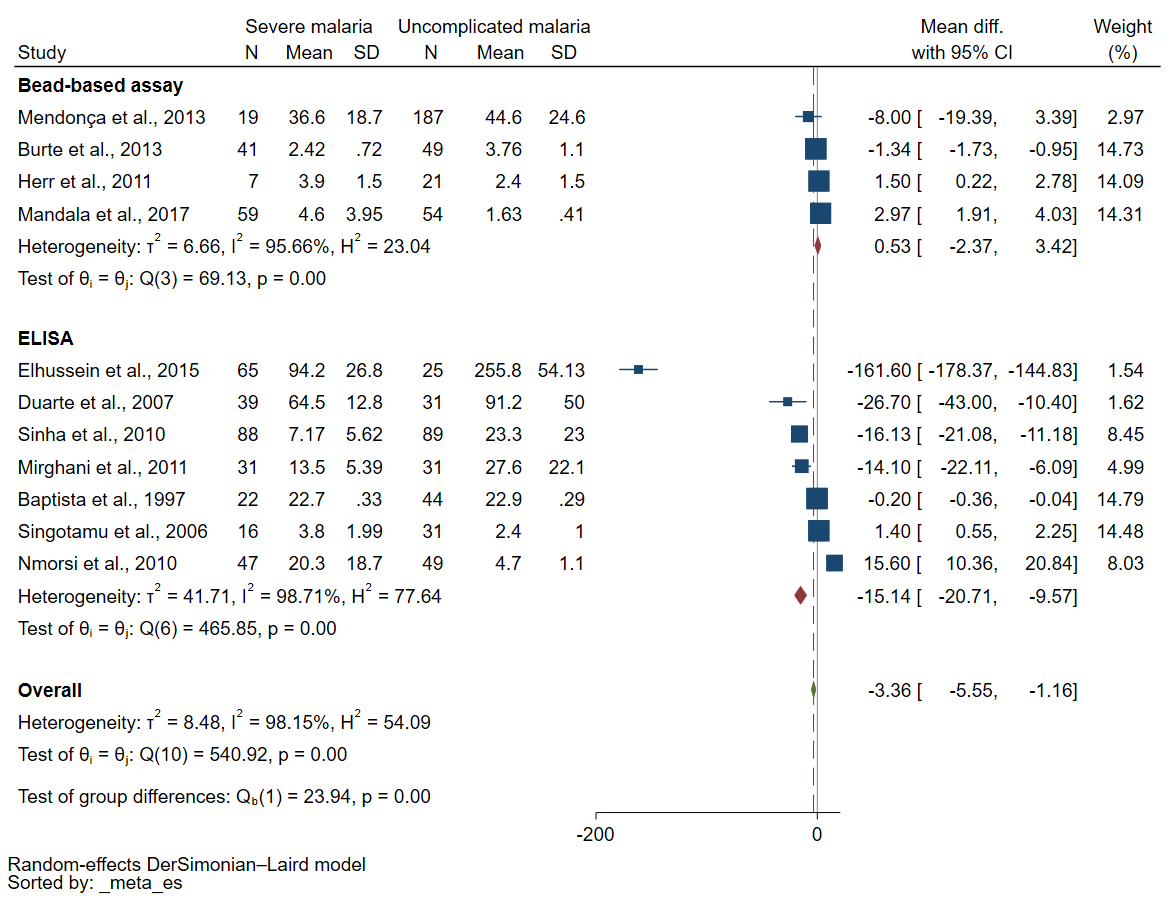

Supplement: Supplementary file 3 — Additional file 3: Figure S3. Mean difference in IL-4 levels between severe malaria and those with uncomplicated malaria patients according to methods for IL-4 measurement. Abbreviation: CI, confidence interval; green diamond symbol, point estimate; solid line in the graph's center at 0 effect size; The dashed red line represents the pooled mean difference between the two groups; the I2 value indicates the degree of heterogeneity; and p = 0.00 or less than 0.05 indicates significant heterogeneity. The weight (%) indicates the contribution of each individual result to the weighted average. IL-4 units in pg/mL. [file 12936_2022_4237_MOESM3_ESM.tif]

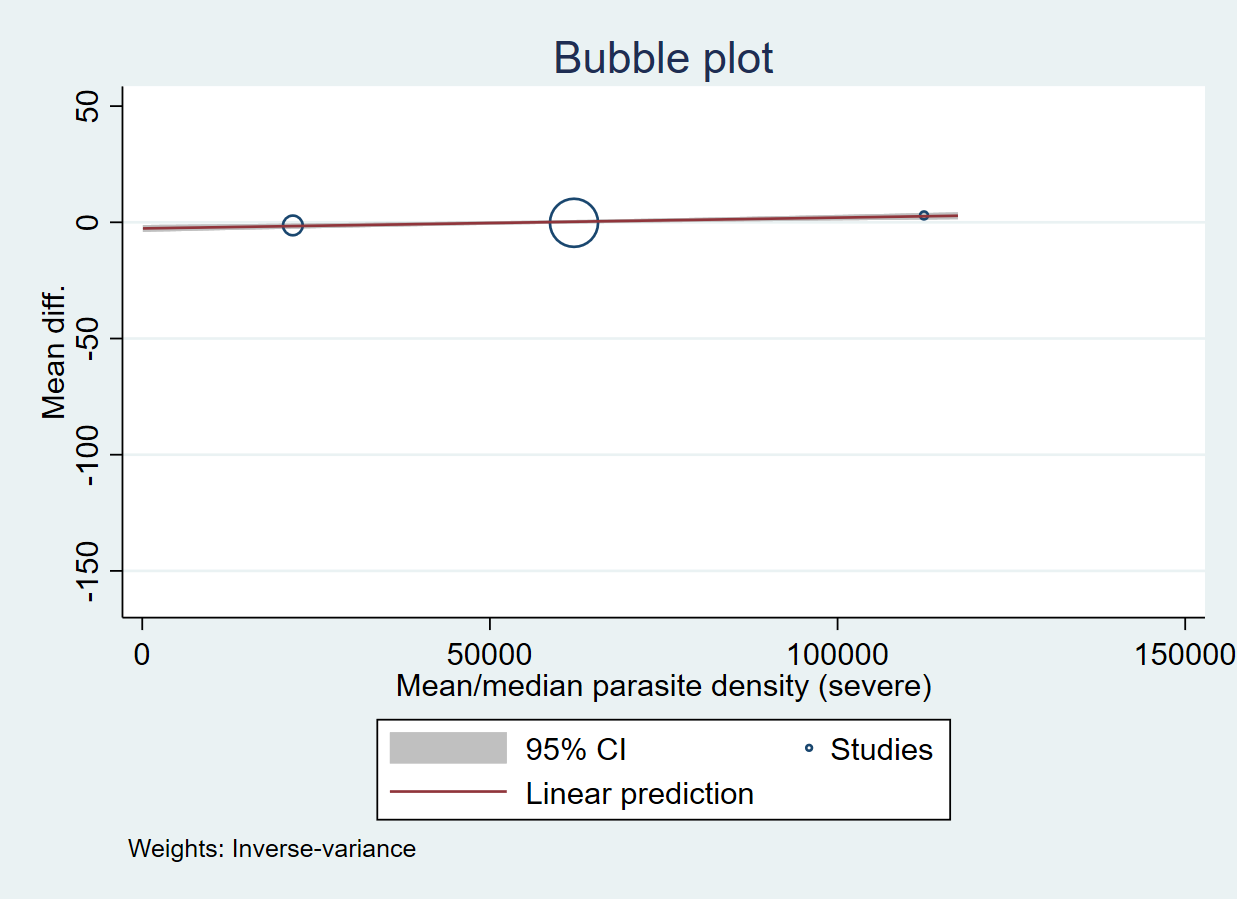

Supplement: Supplementary file 4 — Additional file 4: Figure S4. Bubble plot demonstrating a trend of positive between malaria parasitemia and MD of IL-4 levels. [file 12936_2022_4237_MOESM4_ESM.tif]

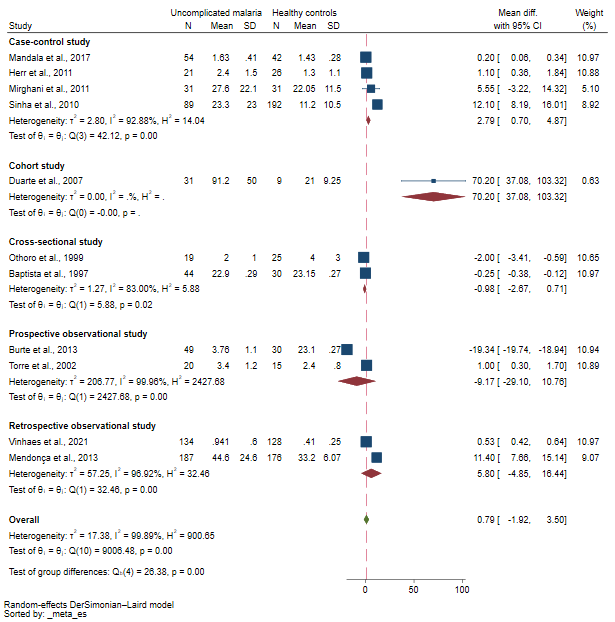

Supplement: Supplementary file 5 — Additional file 5: Figure S5. Mean difference in IL-4 levels between uncomplicated malaria patients and healthy controls according to study designs. Abbreviation: CI, confidence interval; green diamond symbol, point estimate; solid line in the center of the graph at zero effect size, abbreviation; The dashed red line represents the pooled mean difference between the two groups; the I2 value indicates the degree of heterogeneity; and p = 0.00 or less than 0.05 indicates significant heterogeneity. The weight (%) indicates the contribution of each individual result to the weighted average. IL-4 units in pg/mL. [file 12936_2022_4237_MOESM5_ESM.tif]

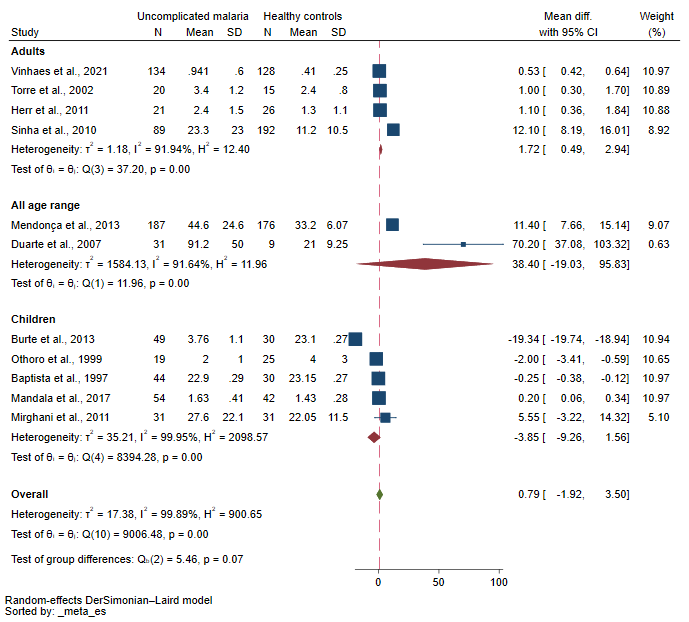

Supplement: Supplementary file 6 — Additional file 6: Figure S6. Mean difference in IL-4 levels between uncomplicated malaria patients and healthy controls according to age groups. Abbreviation: CI, confidence interval; green diamond symbol, point estimate; solid line in the center of the graph at zero effect size, abbreviation; The dashed red line represents the pooled mean difference between the two groups; the I2 value indicates the degree of heterogeneity; and p = 0.00 or less than 0.05 indicates significant heterogeneity. The weight (%) indicates the contribution of each individual result to the weighted average. IL-4 units in pg/mL. [file 12936_2022_4237_MOESM6_ESM.tif]

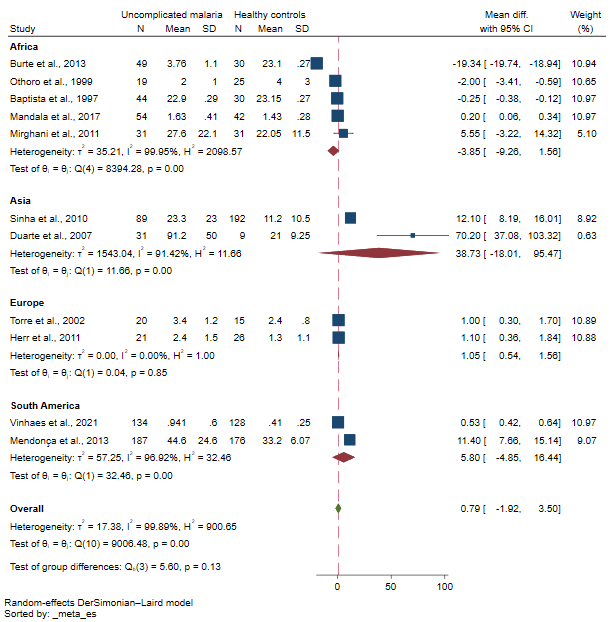

Supplement: Supplementary file 7 — Additional file 7: Figure S7. Mean difference in IL-4 levels between uncomplicated malaria patients and healthy controls according to study sites (continents). Abbreviation: CI, confidence interval; green diamond symbol, point estimate; solid line in the center of the graph at zero effect size, abbreviation; The dashed red line represents the pooled mean difference between the two groups; the I2 value indicates the degree of heterogeneity; and p = 0.00 or less than 0.05 indicates significant heterogeneity. The weight (%) indicates the contribution of each individual result to the weighted average. IL-4 units in pg/mL. [file 12936_2022_4237_MOESM7_ESM.tif]

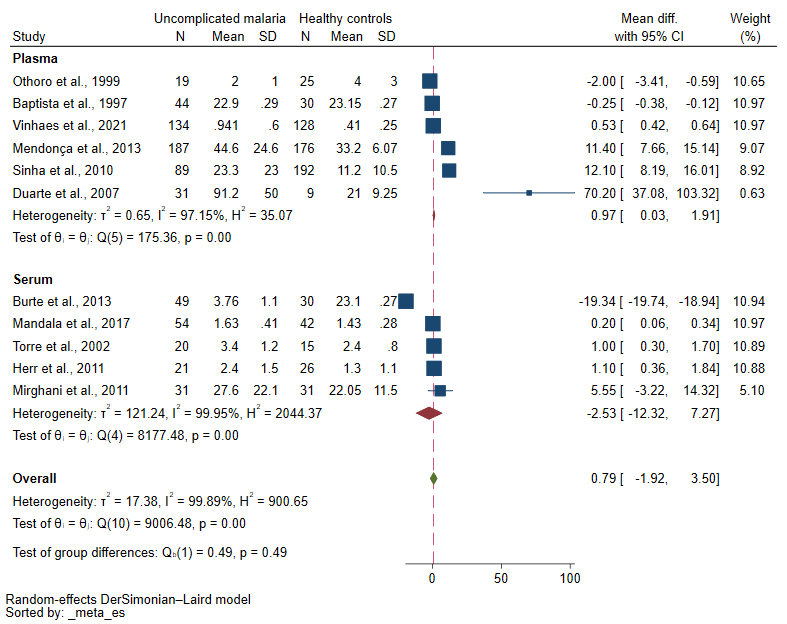

Supplement: Supplementary file 8 — Additional file 8: Figure S8 Mean difference in IL-4 levels between uncomplicated malaria patients and healthy controls according to types of blood samples for IL-4 measurement. Abbreviation: CI, confidence interval; green diamond symbol, point estimate; solid line in the center of the graph at zero effect size, abbreviation; The dashed red line represents the pooled mean difference between the two groups; the I2 value indicates the degree of heterogeneity; and p = 0.00 or less than 0.05 indicates significant heterogeneity. The weight (%) indicates the contribution of each individual result to the weighted average. IL-4 units in pg/mL. [file 12936_2022_4237_MOESM8_ESM.tif]

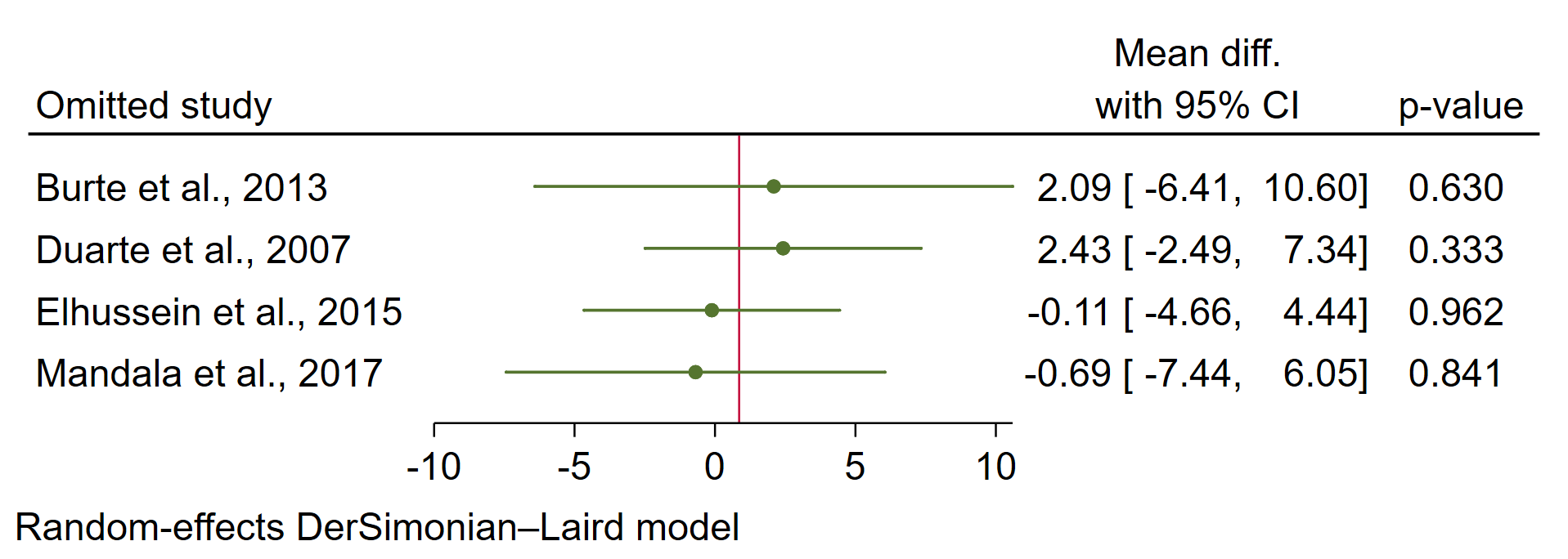

Supplement: Supplementary file 9 — Additional file 9: Figure S9. Sensitivity analysis by leave-one-out method demonstrating the differences in IL-4 levels between cerebral and non-cerebral severe malaria patients by rerunning the meta-analysis and after removing studies. [file 12936_2022_4237_MOESM9_ESM.tif]

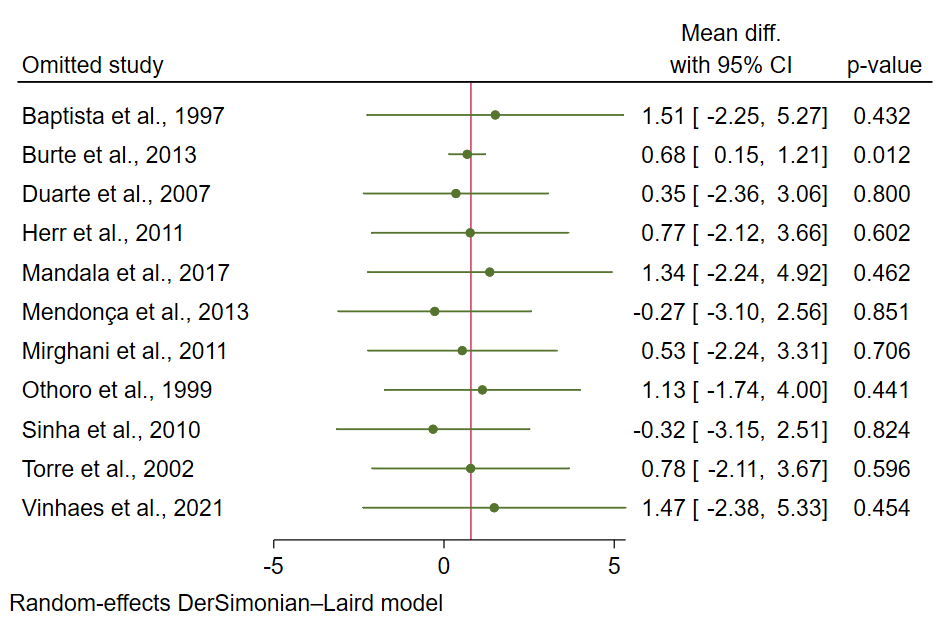

Supplement: Supplementary file 10 — Additional file 10: Figure S10. Sensitivity analysis by leave-one-out method demonstrating the differences in IL-4 levels between uncomplicated malaria patients and healthy controls by rerunning the meta-analysis and after removing studies. [file 12936_2022_4237_MOESM10_ESM.tif]

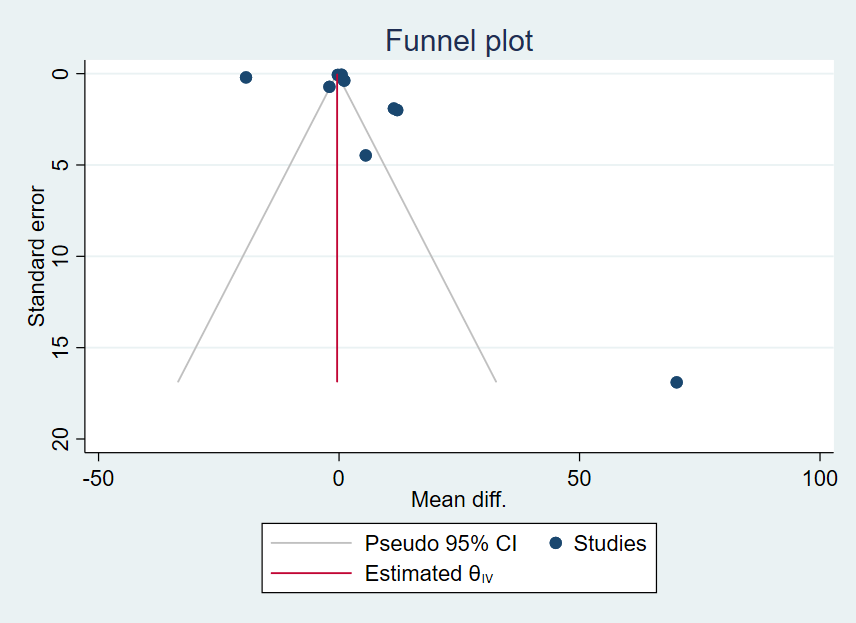

Supplement: Supplementary file 11 — Additional file 11: Figure S11. Funnel plot illustrating the distribution of the mean differences and standard error of the effect size for studies on uncomplicated malaria patients and healthy controls included in the meta-analysis. [file 12936_2022_4237_MOESM11_ESM.tif]

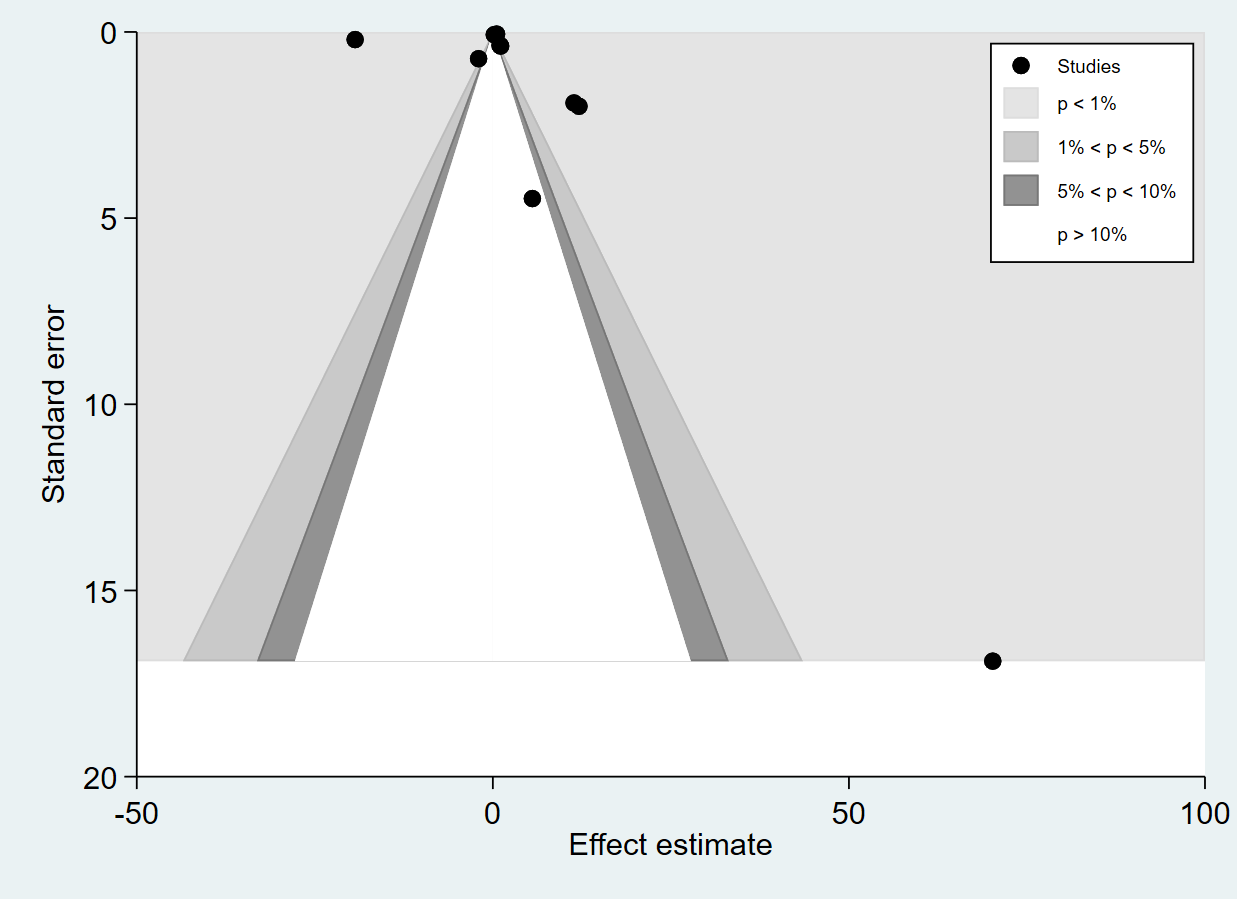

Supplement: Supplementary file 12 — Additional file 12: Figure S12. The contour-enhanced funnel plot revealed that the mean differences of IL-4 levels between uncomplicated malaria patients and healthy controls were located in a statistically significant area (P < 1%, 0.01), indicating that the asymmetry in the funnel plot was caused by publication bias rather than other factors. [file 12936_2022_4237_MOESM12_ESM.tif]
