# Supplementary material for: A systematic review and meta-analysis of blood interleukin-4 levels concerning malaria infection and severity
Source: Malar J. 2022 Jul 12;21:217. doi: 10.1186/s12936-022-04237-z (PMC9277793; doi:10.1186/s12936-022-04237-z)
Supplement: Supplementary file 13 — Additional file 13: Table S1. Search terms. [file 12936_2022_4237_MOESM13_ESM.docx]

**Table S1. Search term**

| **Databases** | **Search terms/Search strategy** | **Date** |
| --- | --- | --- |
| MEDLINE (via PubMed) | 1. interleukin-4 [MeSH]  2. malaria [MeSH]  3. #1 AND #2  Search results: 511 | 21 March 2022 |
| Scopus | ("interleukin-4" OR "interleukin 4" OR IL-4 OR IL4 OR BSF-1 OR Binetrakin OR BCGF-1 OR MCGF-2) AND (malaria OR plasmodium)  Search option: Title, abstract, keywords  Search results: 833 | 21 March 2022 |
| Embase | ("interleukin-4" OR "interleukin 4" OR IL-4 OR IL4 OR BSF-1 OR Binetrakin OR BCGF-1 OR MCGF-2) AND (malaria OR plasmodium)  Search results: 956 | 21 March 2022 |
