# Supplementary material for: A systematic review and meta-analysis of blood interleukin-4 levels concerning malaria infection and severity
Source: Malar J. 2022 Jul 12;21:217. doi: 10.1186/s12936-022-04237-z (PMC9277793; doi:10.1186/s12936-022-04237-z)
Supplement: Supplementary file 15 — Additional file 15: Table S3. Quality of the included studies [file 12936_2022_4237_MOESM15_ESM.docx]

**Table S3. Quality of the included studies**

**Case-control studies**

|  | **Study** | **Score (out of 22)** | **Score (percentage)** | **Quality** |
| --- | --- | --- | --- | --- |
| 1. | Herr et al., 2011 | 19 | 86 | High |
| 2. | Jain et al., 2008 | 19 | 86 | High |
| 3. | Mirghani et al., 2011 | 20 | 91 | High |
| 4. | Mandala et al., 2017 | 20 | 91 | High |
| 5. | Rovira-Vallbona et al., 2012 | 21 | 96 | High |
| 6. | Sinha et al., 2010 | 19 | 86 | High |
| 7. | Tangteerawatana et al., 2007 | 20 | 91 | High |

**Cross-sectional studies**

|  | **Study** | **Score (out of 22)** | **Score (percentage)** | **Quality** |
| --- | --- | --- | --- | --- |
| 1. | Baptista et al., 1997 | 17 | 77 | High |
| 2. | Jakobsen et al., 1994 | 19 | 86 | High |
| 3. | Van den Bogaart et al., 2014 | 19 | 86 | High |
| 4. | Elhussein et al., 2015 | 18 | 82 | High |
| 5. | Othoro et al., 1999 | 17 | 77 | High |
| 6. | Zeyrek et al., 2006 | 18 | 82 | High |
| 7. | Armah et al., 2007 | 22 | 100 | High |
| 8. | Menezes et al., 2018 | 21 | 96 | High |
| 9. | Chaves et al., 2016 | 21 | 96 | High |
| 10. | Ourives et al., 2018 | 21 | 96 | High |
| 11. | Medina et al., 2011 | 20 | 91 | High |

**Prospective observational and cohort studies**

|  | **Study** | **Score (out of 22)** | **Score (percentage)** | **Quality** |
| --- | --- | --- | --- | --- |
| 1. | Duarte et al., 2007 | 19 | 86 | High |
| 2. | Burte et al., 2013 | 20 | 91 | High |
| 3. | Nmorsi et al., 2010 | 15 | 68 | Moderate |
| 4. | Ong’echa et al., 2011 | 19 | 86 | High |
| 5. | Prakash et al., 2006 | 19 | 86 | High |
| 6. | Singotamu et al., 2006 | 16 | 73 | Moderate |
| 7. | Thuma et al., 2011 | 22 | 100 | High |
| 8. | Mendonça et al., 2013 | 21 | 96 | High |
| 9 | Vinhaes et al., 2021 | 21 | 96 | High |
| 10 | Ingoba et al., 2021 | 21 | 96 | High |
| 11. | Davenport et al., 2016 | 20 | 91 | High |
| 12. | Biemba et al., 2000 | 21 | 96 | High |
| 13. | Pinna et al., 2018 | 20 | 91 | High |
| 14. | Thuma et al., 1996 | 19 | 86 | High |
| 15. | Torre et al., 2002 | 18 | 82 | High |
| 16. | Mohapatra et al., 2014 | 16 | 73 | Moderate |
| 17. | Cabantous et al., 2015 | 20 | 91 | High |
| 18. | Okoli et al., 2019 | 19 | 86 | High |

STROBE: Strengthening the Reporting of Observational Studies in Epidemiology
