# Supplementary material for: A systematic review and meta-analysis of blood interleukin-4 levels concerning malaria infection and severity
Source: Malar J. 2022 Jul 12;21:217. doi: 10.1186/s12936-022-04237-z (PMC9277793; doi:10.1186/s12936-022-04237-z)
Supplement: Supplementary file 16 — Additional file 16: Table S4. Meta-regression results. [file 12936_2022_4237_MOESM16_ESM.docx]

**Table S4. Meta-regression results**

| **Comparisons** | *P* value |
| --- | --- |
| **Severe vs uncomplicated malaria**  **Covariates** |  |
| Study designs | < 0.001 |
| Continents | 0.196 |
| *Plasmodium* species | 0.189 |
| Age groups | 0.102 |
| Types of severe complications | 0.029 |
| Malaria parasitemia | < 0.001 |
| Method for IL-4 measurement | < 0.001 |
| Types of blood samples for IL-4 measurement | 0.751 |
| **Cerebral vs non-cerebral malaria**  **Covariates** |  |
| Study designs | 0.278 |
| Continents | 0.126 |
| *Plasmodium* species | NA* |
| Age groups | NA* |
| Malaria parasitemia | 0.295 |
| Method for IL-4 measurement | 0.715 |
| Types of blood samples for IL-4 measurement | 0.126 |
| **Uncomplicated malaria vs healthy controls**  **Covariates** |  |
| Study designs | 0.002 |
| Continents | 0.004 |
| *Plasmodium* species | 0.247 |
| Age groups | 0.002 |
| Malaria parasitemia | 0.632 |
| Method for IL-4 measurement | 0.108 |
| Types of blood samples for IL-4 measurement | 0.013 |

*NA: not applicable due to a low number of studies for calculation
